# A Novel Hardware Systolic Architecture of a Self-Organizing Map Neural Network

**DOI:** 10.1155/2019/8212867

**Published:** 2019-04-01

**Authors:** Khaled Ben Khalifa, Ahmed Ghazi Blaiech, Mohamed Hédi Bedoui

**Affiliations:** ^1^University of Sousse, Higher Institute of Applied Sciences and Technology of Sousse, Sousse, Tunisia; ^2^University of Monastir, LR12ES06-Laboratory of Technology and Medical Imaging, Monastir, Tunisia

## Abstract

In this article, we propose to design a new modular architecture for a self-organizing map (SOM) neural network. The proposed approach, called systolic-SOM (SSOM), is based on the use of a generic model inspired by a systolic movement. This model is formed by two levels of nested parallelism of neurons and connections. Thus, this solution provides a distributed set of independent computations between the processing units called neuroprocessors (NPs) which define the SSOM architecture. The NP modules have an innovative architecture compared to those proposed in the literature. Indeed, each NP performs three different tasks without requiring additional external modules. To validate our approach, we evaluate the performance of several SOM network architectures after their integration on an FPGA support. This architecture has achieved a performance almost twice as fast as that obtained in the recent literature.

## 1. Introduction

Various hardware implementations of self-organizing map (SOM) neural networks on hardware circuits have been presented in the literature. They may be divided into two main categories. Firstly, analogical implementations on dedicated integrated circuits have been designed [1–5]. These supports are technically limited as they lack precision. Their performance greatly depends on the used technology. Second, digital implementations on ASIC circuits (neuroprocessors) have also been designed [6–10]. This is now the most used category of VLSI for neuromimetic algorithms.

Indeed, all these integration approaches have been generally widely used because they have the advantage of higher accuracy, better repeatability, lower noise sensitivity, better testability, and greater flexibility and compatibility with other types of neuroprocessors (NPs) constituting the neural network. However, the configuration of these systems is too complex for users who are not specialist, and it does not offer the reconfigurability by the users.

The above shortcomings of both types of implementation devices may be avoided, thanks to reprogrammable circuits, such as field-programmable gate arrays (FPGA). These circuits offer high-performance, high speed, and low cost, especially if we target prototyping applications and high-capacity programmable logic solutions that enhance design. These circuits provide also low power consumption design. The availability of material on chip enables the designer to imagine a parallel SOM architecture. Configurable hardware appears well adapted to obtain efficient and flexible neural network implementation. Several SOM implementations on FPGA supports have been proposed [11–18]. Indeed, Porrmann et al. in [11] successfully implemented, on a Virtex FPGA support, a reconfigurable SIMD architecture of an SOM network formed by a processing element (PE). The computation was performed exclusively in parallel between PEs of the same type that formed the system and the input vector. The suggested architecture was composed, in addition to the PE modules, of external control blocks and memory to save the weights of the neurons forming the SOM network. In [12], Tamukoh and Sekine put forward a dynamical SOM hardware architecture. The latter utilized flexible and reconfigurable PEs according to the number of neurons and the size of the input vector. In this work, the exploitation of the dynamic reconfiguration of FPGA circuits provided more flexibility, but at the detriment of performance. Ramirez-Agundis et al. in [13] proposed a massively parallel hardware solution for various neuron numbers on the SOM output map (16, 32, 64, 128, and 256 neurons). The authors evaluated their architectures on a video coding application. The solution suggested by the authors was structured around neural computation modules and a comparator whose resolution depended on the topology of an SOM network to be integrated. In [14], the authors presented an SOM-network implementation on an FPGA with a new asynchronous and parallel neighbourhood approach, based on the triangular neighbourhood function method. This approach was needed to calculate the distance between the winner neuron and its neighbouring neurons. The proposed architecture was not very efficient since it had a very complex neighbourhood-control module that used a large number of clock cycles, which consequently slowed down the overall performance of the SOM network. In [15], Kurdthongmee put forward an approach to accelerate the learning phase of an SOM hardware architecture (called K-SOM) by evaluating the mean square error (MSE) after image color quantization. The author used a single 16 × 16 map to evaluate this approach on different images varying from 32 × 32 to 512 × 512 pixels. The suggested approach was validated on a Xilinx Virtex-2 FPGA, providing real-time performances for image sizes up to 640 × 480 pixels. The K-SOM experimental results were more efficient than other approaches in terms of video rate frame and MSE: about 50% faster in the frame rate and 25% lower in the MSE. In [16], the same author put forward a new method to locate the winning neuron in an SOM network in one clock cycle. This method was based on the exploitation of memory formed by the neuron indices that were addressable by distance values. This new approach allowed reaching a maximal operating frequency of 47 MHz and a number of frames per second (fps) equal to 22. For image compression, the authors in [17] successfully integrated a completely parallel SOM on an FPGA circuit using a shared comparator to exploit the parallelism between different neuroprocessors. In [18], the authors have proposed a new scalable and adaptable SOM network hardware architecture. The suggested architecture would permit dynamically modifying the SOM network pattern only by reconfiguring each neuron. This scalability was obtained by separating the computational layer with neurons from the communication layer. Moreover, scalability is used to provide data exchange mechanisms between neurons by interposing routing modules based on the Network-on-Chip (NoC) technique. Despite the modularity and flexibility of this architecture, it had poor performances in hardware resources as well as execution time.

Most of these approaches depend on the SOM-architecture configuration, such as the number of input vector elements, output layer size, time constraints, and memory requirements. Almost all these parameters are specified during the design phase of the SOM.

Also, the internal architecture of (NP) computation units, presented in the literature, requires other additional modules necessary for (i) the localization of the neuron (called “winning neuron”), at the output layer of SOM, closest to the input vector *X*, using a shared comparator which consumes many blocks and whose complexity increases quadratically with the number of neurons, (ii) the weight adaptation of the winning neurons and their neighbours, and (iii) the global control of the SOM network. As a result, this makes it impractical to use these approaches for integrating large networks.

To overcome these limits, we propose to integrate a new neuroprocessor architecture ensuring the three specific tasks to the SOM network operation: (i) the calculation of the Euclidean distance, (ii) the extraction of the minimal distance, and (iii) updating the weights of the winning neuron as well as of these neighbours. This solution enables us to reduce the time and the number of connections between the various SOM modules by eliminating the shared comparator and replacing it with local comparators for each neuroprocessor.

In order to make our architecture more flexible and efficient in terms of clock cycles, we will adopt a systolic architecture. These architectures are based on a concept in which a single data path traverses all neural PEs and is extensively pipelined. This approach makes it possible to reduce the number of communication paths in the network and simultaneously reduce the number of cycles required to classify a data vector. This will provide a very high clock frequency. For example, we cite Ienne et al.'s work in [19] who implemented two architectures of the SOM algorithm using two-dimensional systolic approaches. They used the MANTRA I platform to validate their approaches. The achieved performance was about 13.9 MCUPS in the learning phase. Another systolic implementation of a 1D-SOM was proposed in [20] on an FPGA. The performance of the solution is 3208 MCUPS.

Consequently, this solution will make our architectures generic and flexible during the design phase, as it allows checking implementation constraints (embedded memory size, arithmetic operator resolution, and power consumption) and rapidly adapting to parameters related to the topology of the integrated SOM neural network. The main contributions of this work are as follows:

- Implementing a new architecture with systolic interconnections, based on the use of configurable neuroprocessors, each of which provides neural calculation and local comparison
- Proposing a new local neighbourhood function for each neuroprocessor, based on the shift principle, while taking into account the neuron position as regards the winning neuron and considering the number of epochs used during learning
- Proposing a pipelined scheduling mode for searching the minimum distance and the identifier of the winning neuron in a systolic way

This article is organized as follows. In Section 2, we present the SOM Kohonen model with emphasis on its algorithmic aspect. In Section 3, we highlight the problems solved by our approach. In Section 4, we detail the proposed parallel architecture and the formalism adopted to estimate the execution time.  Section 5 presents the internal architecture of the nodes for the SOM model implementation. The obtained results are provided in Section 6. In Section 7, we present a color quantization and image compression application to validate the SSOM architecture.

## 2. The Self-Organizing Map (SOM)

SOMs are artificial oriented neural networks characterized by their unsupervised learning, as defined in [21]. Technically, these neural models perform a “vector quantification” of the data space by adopting a discretization of the space by dividing it into zones, each represented by a significant point called the referent vector or codebook.

Architecturally, SOMs are made up of a grid (usually one or two-dimensional). In each node of this grid, we find a “neuron”. Each neuron is linked to a referent vector, responsible for an area in the data space (also called the input space).

In an SOM, the reference vectors provide a discrete representation of the input space. They are positioned in such a way that they retain the topological shape of the input space of size Dim_*x*_. By keeping neighbourhood relationships in the grid, they allow an easy indexation formed by *P* ∗ *Q* neurons where *P* and *Q* are, respectively, the number of columns and rows (via coordinates in the grid). This is useful in various areas, such as texture classification, interpolation between data, visualization of multidimensional data, etc.

The SOM learning algorithm is competitive and runs in two steps: selecting the winning neuron and then updating the weights of the winning neuron and its neighbours.

For each input vector $\vec{X}=\left\{x_{0},x_{2},\ldots ,x_{{\text{Dim}}_{x}-1}\right\}\in R^{{\text{Dim}}_{x}}$ selected at time *t*, the Euclidean distance *d*_(*l*, *k*)_ to all weight vectors $\vec{M_{l,k}}=\left\{\mu_{\left(l,k\right),0},\mu_{\left(l,k\right),1},\ldots ,\mu_{\left(l,k\right),{\text{Dim}}_{x}-1}\right\}\in R^{{\text{Dim}}_{x}}$, where (*l*, *k*) are the coordinates of each neuron, is computed as follows:

(1) $$\begin{array}{c} d_{\left(l,k\right)}=\left\|\vec{X}-\vec{M_{l,k}}\right\|=\sqrt{\overset{N-1}{\underset{j=0}{\sum }}{\left(x_{j}-\mu_{\left(l,k\right),j}\right)}^{2}}. \end{array}$$

Subsequently, the neuron with the smallest distance, which is called the winner neuron *N*_c_, is determined as follows:

(2) $$\begin{array}{c} N_{\text{c}}=\arg\min_{l,k}\left\{\left\|\vec{X}-\vec{M_{l,k}}\right\|\right\}\text{with 0}\leq l\leq P-1\text{ and }0\leq k\leq Q-1. \end{array}$$

After selecting the winner node, the weight vectors associated to this node and its neighbours, located in a defined area around the node *N*_c_, are adjusted in such a way that their profile is close to the input data. This adjustment of weights, which characterizes the unsupervised learning of the model, can be described by

(3) $$\begin{array}{c} \vec{M_{l,k}}\left(t+1\right)=\vec{M_{l,k}}\left(t\right)+h_{c,l,k}\left(t\right)\left(\vec{X}\left(t\right)-\vec{M_{l,k}}\left(t\right)\right), \end{array}$$

where *h*_*c*,*l*,*k*_(*t*) is the neighbourhood function whose value represents the strength of the coupling between two nodes during the learning process. This function depends on the position of the neuron of coordinates (*l*, *k*) with respect to the winner's one and on the epoch number *t* representing the number of learning iterations.

## 3. SOM Based on Systolic Architecture

The proposed architecture is formed by two parts. The first part concerns the computation of the Euclidean distances between the input vector and all the neurons forming the SOM network output layer. It is worth noting that computing all distances will be done in parallel at the same time for all neurons.

The second part concerns the extraction of the minimum distance as well as the identifier corresponding to the winning neuron. In this part, we will adopt a systolic formalism based on the pipeline transmission of distances and identifiers between the neighbouring neurons.

**Figure 1:**
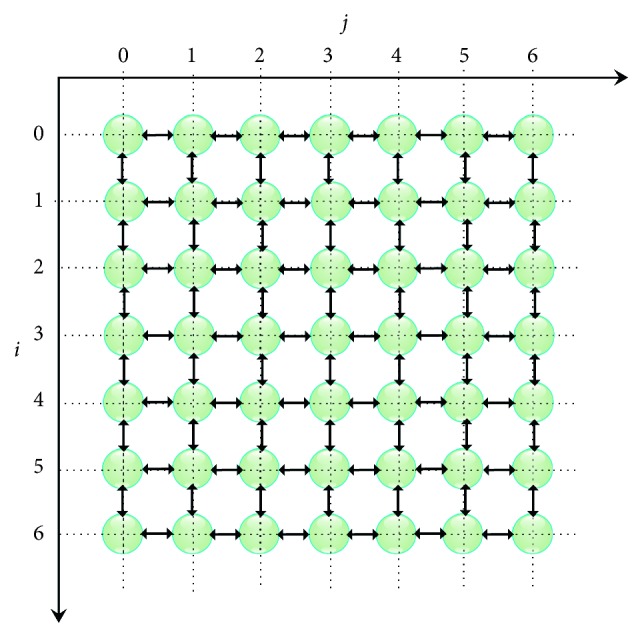
Establishment of connections between SOM nodes (*P*=7 and *Q*=7).

This architecture, called systolic-SOM (SSOM), is formed by a set of the same nodes placed in a two-dimensional space. The direction of data exchange between nodes can perform the entire neural algorithm in its decision and learning phases. In the decision phase, the intermediate comparison results already processed in each node are propagated in parallel to all the neighbouring neurons that surround it directly, to extract the global winner node identifier (Figure 1). In the following section, we present the formalism adopted for the implementation of the various generic SOM architectures. This formalism consists in presenting our neural network as a data-flow graph composed of nodes and arcs.

**Figure 2:**
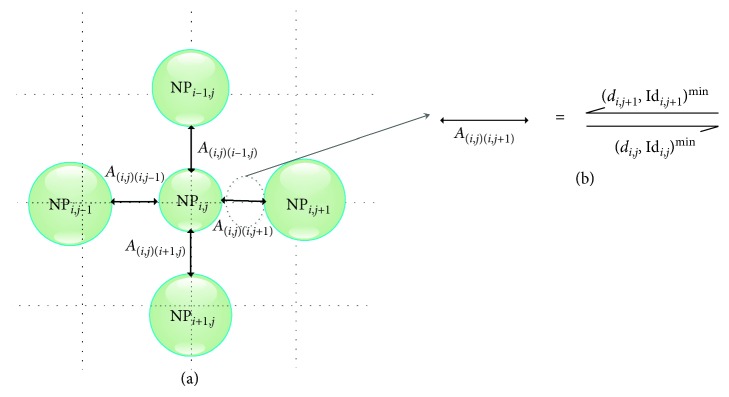
Example of interconnection between a NP_*i*,*j*_ node and its neighbours.

The SSOM network is formed by a set of elementary NPs, each of which emulates the neuron operations. These NPs are interconnected within a network that is modeled by an oriented and symmetrical graph *G*(*O*, *A*). This graph is formed by nodes and arcs. Each node, noted as NP_*i*,*j*_ ∈ *O*, represents an elementary neuroprocessor with *i* as the line index and *j* as the column index. An arc noted *A*_(*l*_*i*_, *l*_*j*_)(*m*_*i*_, *m*_*j*_)_ ∈ *A* defines a bidirectional communication link between two nodes, NP_*l*_*i*_,*l*_*j*__ and NP_*m*_*i*_,*m*_*j*__ ∈ *O* with respective coordinates (*l*_*i*_, *l*_*j*_) and (*m*_*i*_, *m*_*j*_). Indeed, Figure 2(a) illustrates an example of the interconnection between an NP_*i*,*j*_ and its four nearest neighbours in the graph *G*. Noting that each of the NP_*i*,*j*_ is interconnected to all of these neighbours through bidirectional arcs that simultaneously broadcast and receive the minimum distances as well as the identifiers of the corresponding nodes (Figure 2(b)).

The basic idea of the proposed architecture is to perform a neural computation in a competitive way at each node, as described in the introduction. In addition, each of the nodes forming the SSOM will ensure three local calculation phases necessary for the overall execution of the SOM: (i) computing the distance between a vector and the various weights of the neurons in the output grid, which will be executed in parallel on all neurons, (ii) extracting the winning neuron, which will run in a systolic manner, and (iii) updating the winning neuron and these neighbours in parallel for all neurons.

During the minimal distance extraction and the winning neuron identifier localization phases, *p*_*i*_ scheduling processes are established. During these processes, the minimal distances and identifiers (*d*_*l*,*k*_, Id_*l*,*k*_)^min^ retrieved at the output of each node will propagate to all neighbouring nodes. Note that, on the one hand, the number of *p*_*i*_ processes will depend exclusively on the size of the SOM network and that it is equal to *P* + *Q* − 1.

**Figure 3:**
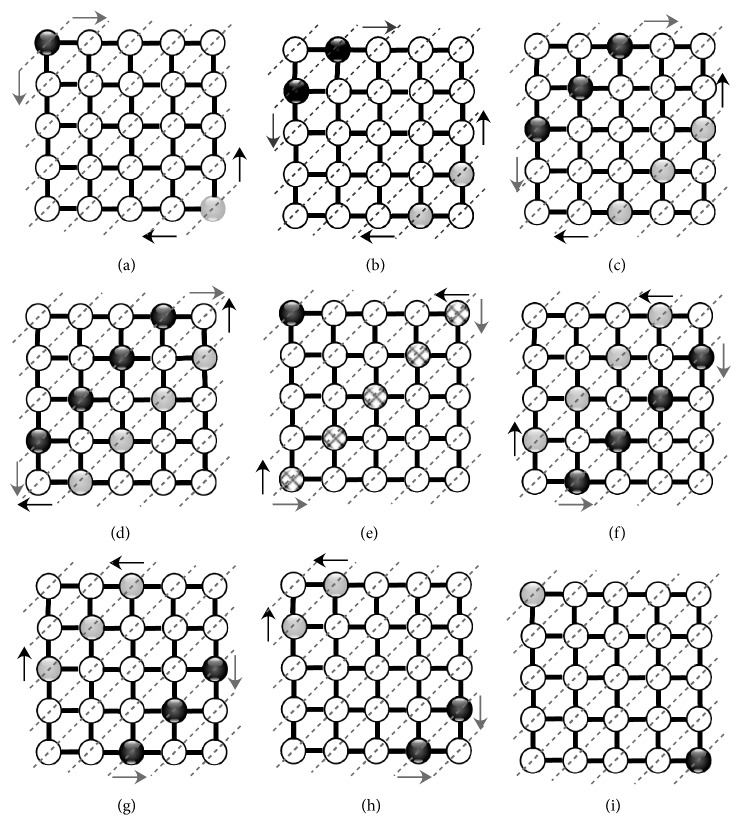
Process scheduling during propagation phase (*P*=5 and *Q*=5). (a) Process *P*_1_. (b) Process *P*_2_. (c) Process *P*_3_. (d) Process *P*_4_. (e) Process *P*_5_. (f) Process *P*_6_. (g) Process *P*_7_. (h) Process *P*_8_. (i) Process *P*_9_.

In Figure 3, the black and grey nodes illustrate an example of the information propagation (distances and IDs) located in both corners of the SOM map.

Indeed, for instance in Figure 3, we differentiate nine processes of pipelined distance propagation between the various neuroprocessors (of coordinates (*l*, *k*)) in a systolic way.  Table 1 shows all output torques (*d*_*l*,*k*_, Id_*l*,*k*_)^min^ representing the minimum distances at each neuroprocessor as well as the corresponding Id. For example in Table 1, the neuron of coordinate (4, 4) disposes of the minimum distance which is equal to 2. Thus, after nine processes (*P*_9_), all neurons will locate the minimum distance without using additional lines to backpropagate the distance and identify it, as it is the case for [19, 20].

## 4. Functional Description of SSOM Architecture

The operation of each NP defined in the SSOM is decomposed into three tasks: distance computation, winning neuron extraction, and updating the winning neuron and its neighbour's weights.

### 4.1. Local Distance Computation

During this phase, the NP computes the Euclidean distance between the input vector $\vec{X}$ and the weight vector $\vec{M}$ corresponding to the relevant neuron according to equation (1). Obviously, each calculated distance in the SOM is positive *d* ≥ 0. Thus, for any two neurons, *n*_1_ and *n*_2_, if *d*_*n*_1__ < *d*_*n*_2__, then *d*_*n*_1__^2^ < *d*_*n*_2__^2^. For this reason, both *d* and *d*^2^ lead to the same result in the process of identifying the winning neuron. Moreover, measure *d*^2^ is often favored over measure *d* because it allows omitting the rooting operation and therefore decreases the computational complexity of the SOM algorithm [22, 23].

Measure *d*^2^ is calculated throughout this work, and for a neuron at position (*l*, *k*), we get

(4) $$\begin{array}{c} {\left(d_{\left(l,k\right)}\right)}^{2}=\left\|\vec{X}-\vec{M_{l,k}}\right\|=\overset{N-1}{\underset{j=0}{\sum }}{\left(x_{j}-\mu_{\left(l,k\right),j}\right)}^{2}. \end{array}$$

This phase is carried out in parallel with all other neurons in the SSOM grid. During the same phase, the $\vec{\Delta M_{i}}\left(t\right)$ is prepared with $\vec{\Delta M_{i}}\left(t\right)=\vec{X}\left(t\right)-\vec{M_{i}}\left(t\right)$ and will only be used (during the update phase) in the case where the neuron in the question is declared the winner or neighbour of the winner.

### 4.2. Extraction and Updating of the Winning Neuron

We define the winning neuron as the neuron closest to the input vector $\vec{X}$. Indeed, each node NP_*i*,*j*_ delivers at its output the minimal distance squared (*d*_*i*,*j*_^min^)^2^ (equation (5)) and Id_*i*,*j*_^min^ (equation (6)) corresponding to the identifier of the neuron that provides the minimal distance. Note that (*d*_*i*,*j*_^min^)^2^ represents the value of the minimal distance squared between the proper distance squared (*d*_(*l*, *k*)_)^2^ of the node in question and other delivered from the neighbours nodes:

(5) $$\begin{array}{c} {\left(d_{i,j}^{\min}\right)}^{2}=\min\left(\text{distance}\left(\text{Neighb}\left({\text{NP}}_{i,j}\right)\right)\right), \end{array}$$

(6) $$\begin{array}{c} {\text{Id}}_{i,j}^{\min}=\arg\left({\left(d_{i,j}^{\min}\right)}^{2}\right). \end{array}$$

Thus, each NP_*i*,*j*_ node will propagate the (*d*_*i*,*j*_^min^)^2^ distance and the Id_*i*,*j*_^min^ identifier through a bus to the successor NP_*i*′,*j*′_ node. This operation is executed until achieving a stabilized state at each node. Each node provides the minimal squared distance (*d*^min^)^2^ as well as the identifier of the wining node Id_c_ over the SOM network. Towards the end, the winning neuron and its neighbours will be updated using the neighbourhood functions already calculated during the local distance computation.

## 5. Implementation of SSOM Network

To integrate the SSOM network architecture, we propose to use NP modules where each emulates the operation of a neuron in SOM. Thus, all NPs forming SSOM will be placed in an array structure.

### 5.1. Internal NP Architecture

Each NP is composed by two basic modules: the processing unit, called SOMPE, and the comparator unit with five inputs for the minimal distance extraction. SOMPE ensures, on the one hand, the calculation of the Euclidean distance between the *X* vector and the $\vec{M}$ weight of the corresponding neuron and, on the other hand, the weight update if the concerned neuron is considered a winner or neighbour of a winning neuron. The comparator gives the minimal distance as well as the identifier of the corresponding node to its output.

### 5.2. SOMPE Architecture

**Figure 4:**
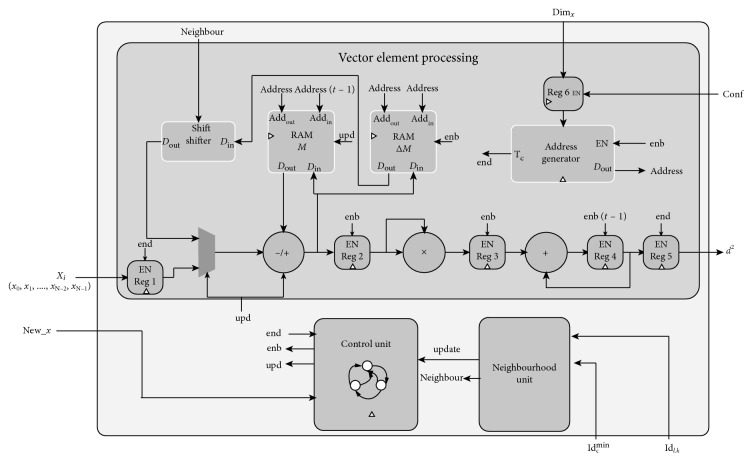
SOMPE architecture.

Figure 4 illustrates the SOMPE architecture. It is composed of three basic units.

#### 5.2.1. Vector Element Processing Unit

This unit executes the two neuron-computation phases: decision and weight updating. The vector element processing (VEP) uses basic operators (a subtractor, a multiplier, and an adder) to calculate the Euclidean distance between the *x*_*k*_ elements (with a, *k*=0,…, Dim_*x*_ − 1) of a vector $\vec{X}$ and an $\vec{M}$ weight vector that corresponds to a specific node. The *x*_*k*_ elements are serially sequenced at the SOMPE input (element by element).

In addition to the distance computation, VEP is composed of two other blocks. A first block is necessary to the weight update preparation. The second block precalculates the vector values $\vec{\Delta M}\left(t\right)=\vec{X}\left(t\right)-\vec{M}\left(t\right)$ in parallel with the distance calculation phase and stores them in the RAM memory. The size of this memory depends on the number of the elements of the weight vector and on the accuracy of each element in terms of bit number.

#### 5.2.2. Neighbourhood Unit (NU)

This unit controls the execution of the operation $\vec{M_{l,k}}\left(t\right)+h_{c,l,k}\left(t\right)\cdot \vec{\Delta M_{l,k}}\left(t\right)$ necessary to update the weights of the winning neuron and its neighbours. *h*_*c*,*l*,*k*_(*t*) is the neighbourhood function already presented in Section 2 (equation (3)) and $\vec{\Delta M_{l,k}}\left(t\right)$ is already calculated by VEP. For the integration of the update operation, recent work in the literature [8, 10, 12, 17] has used multiplication operators and memory modules to store the results. This is generally costly in logic resources. To overcome this limitation while removing the multiplication operations between *h*_*c*,*l*,*k*_(*t*) and $\vec{\Delta M_{l,k}}\left(t\right)$, we use the bit shift to the right operators. In fact, an S-bit shift of the $\vec{\Delta M_{l,k}}\left(t\right)$ value corresponds to its multiplication by *h*_*c*,*l*,*k*_(*t*)=1/2^*s*^. Therefore, to remain under the same conditions presented in Section 2,

(7) $$\begin{array}{c} h_{c,l,k}\left(t\right)=\left\{\begin{array}{cc} \frac{1}{2^{S}}=\frac{1}{2^{r_{l,k}+\beta \left(t\right)}}, & \text{if }r_{l,k}\leq R\left(t\right),\\ 0, & \text{otherwise,} \end{array}\right. \end{array}$$

(8) $$\begin{array}{c} r_{l,k}=\left|l-c_{l}\right|+\left|k-c_{k}\right|, \end{array}$$

(9) $$\begin{array}{c} \beta \left(t\right)=\text{round}\left(\frac{t}{L*K*10}\right). \end{array}$$

**Figure 5:**
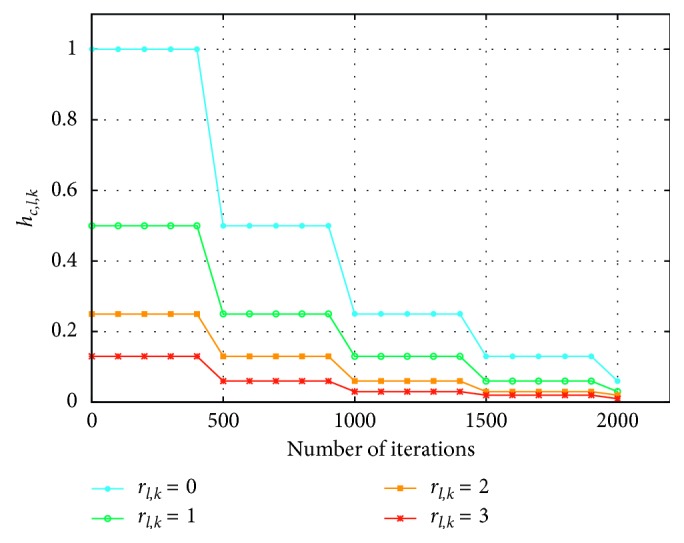
Variation in value of *h*_*c*,*l*,*k*_(*t*) as a function of *r*_*l*,*k*_ and the number of epochs (iterations).

Indeed, the *S* number of shifts is determined according to *r*_*l*,*k*_, which represents the neighbourhood radius between the neuron of coordinates (*l*, *k*) and the winner neuron of coordinates (*c*_*l*_, *c*_*k*_) (equation (7)). It is also determined as regards the learning phase progress *β*(*t*) related to the *t* epoch number, which is defined by equation (9). Note that *β*(*t*) is initially equal to zero. *R*(*t*) represents the maximal radius that is initially equal to (*P* + *Q*). The value of *R*(*t*), which defines the variation in the maximal neighbourhood radius value as a function of the number of epochs, varies according to the *β*(*t*) value, as indicated by equation (10). The function *R*(*t*) decreases until stabilizing at a value equal to one, which represents the neighbour radius, after a defined number of iterations. This is intended to minimize the effect of *h*_*c*,*l*,*k*_ on the neighbours far away from the winning neuron (Figure 5).

(10) $$\begin{array}{c} R\left(t\right)=\left\{\begin{array}{cc} \left(P+Q\right)-\beta \left(t\right), & \text{if }P+Q>\beta \left(t\right),\\ 1, & \text{otherwise}. \end{array}\right. \end{array}$$

#### 5.2.3. Control Unit (CU)

This is based on a finite state machine (FSM) included in SOMPE. The transition of this FSM from one state to another is controlled by the two signals which, respectively, control the decision and updating phases.

### 5.3. Comparator Architecture Block

For the extraction of the minimal distance *d*_*l*,*k*_^min^ as well as the identifier Id_*l*,*k*_^min^ of the corresponding node, we use a modular comparator *C*_5_ with 5 inputs, each of which is represented by the pair (*d*_*i*,*j*_, Id_*i*,*j*_). The internal architecture of this block is formed by cascaded elementary comparators.

### 5.4. Neuroprocessor Integration

**Figure 6:**
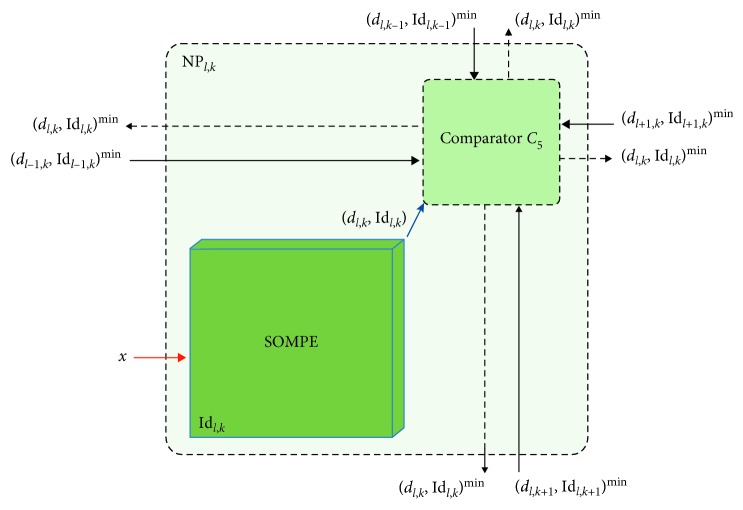
Internal NP architecture.

For the SSOM-architecture implementation, we will assign to each node of the connection grid an NP (Figure 6). The internal architecture of each of these NPs depends on the relevant node position. The NP is composed, in addition to its SOMPE module, of a comparator of type *C*_5_ which receives the distance coming from its neighbours and which will compare it with its own distance (calculated by SOMPE).

## 6. Results of Implementation of SSOM Architecture

### 6.1. Resource Evaluation

The SSOM design has been developed using VHDL code and has been synthesized with Xilinx ISE Design Suite 14.4 tool. The constructed architecture has been performed for XC7VX485t-2FFG1761 Xilinx FPGA family devices.

**Figure 7:**
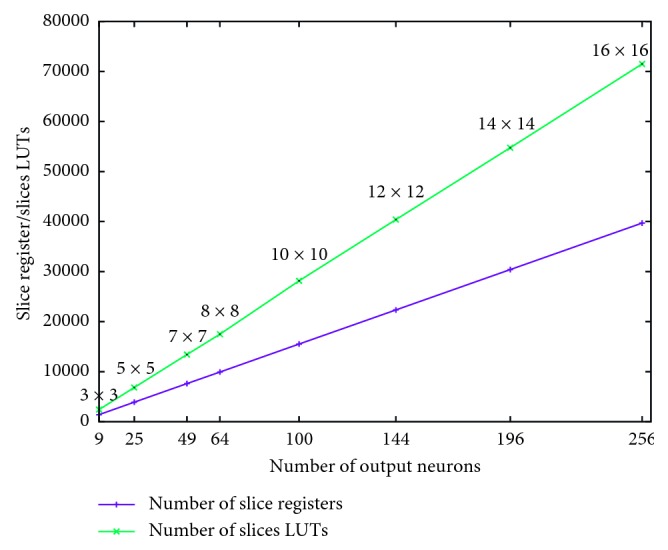
Variation in number of hardware resources (slice and LUT) for different SOM topologies with Dim_*x*_ = 32.

Figure 7 shows the number of hardware resources (slices and LUTs) for various SSOM topologies with the same dimension of the input vector (Dim_*x*_ = 32). We note that the number of different resources varies linearly according to the number of neurons on the output layer. This is simply because the number of operators used for setting different topologies also varies linearly as a function of the neurons number on the output layer.

Moreover, unlike the architectures presented in the literature, our solution is clocked with a maximal frequency equal to 290 MHz whatever the SSOM topology.

### 6.2. Performance Evaluation

For the evaluation and analysis of the temporal SSOM-architecture performance, we opt for several SOM network topologies. The architecture implantation parameters are provided in Table 2. Table details the architecture performance during the distance calculation phases: propagation, competition, and updating. Furthermore, this table shows the overall performance of the entire architecture.

According to Table 3, the time required for the decision phase *t*_c_ is equal to *t*_d_+*t*_p_, where *t*_d_ is the time required for the distance calculation and *t*_p_ corresponds to the propagation time of the minimal distance through the longest path. In addition, the overall learning time *t*_l_ during a single iteration is equal to the sum of the decision time *t*_c_ while adding the weight adapting time *t*_a_. For example, with a 16 × 16-SOM topology, the number of cycles corresponding to the decision and learning phases is equal to 65 and 99 clk, respectively.

In general, the performance of the SOM neural network is evaluated by MCUPS for the leaning phase (equation (11)) and million connections per second (MCPS) for the recall phase (equation (12)). All the parameters of the two equations are presented and explained in Table 1.

(11) $$\begin{array}{c} \text{MCUPS}=\frac{P\cdot Q\cdot {\text{Dim}}_{x}\cdot \text{Freq}}{t_{\text{l}}}, \end{array}$$

(12) $$\begin{array}{c} \text{MCPS}=\frac{P\cdot Q\cdot {\text{Dim}}_{x}\cdot \text{Freq}}{t_{\text{c}}}. \end{array}$$

**Figure 8:**
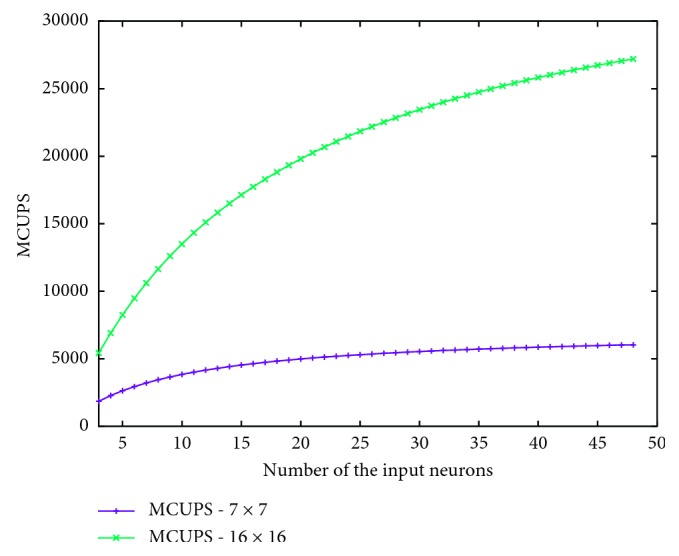
Variation in MCUPS depending on number of neurons on input layer with 7 × 7 and 16 × 16 neurons on the output layer.

Figure 8 illustrates the MCUPS variation according to the number of input neurons for a fixed topology with, respectively, 7 × 7 and 16 × 16 neurons on the output layer. We firstly note that the variation in both parameters increases nonlinearly (the MCUPS value reaches 24,000 for a topology of 16 × 16 neurons and 32 inputs). On the one hand, a high number of neurons in the input layer have a low impact on the variation in MCUPS. On the other hand, a low number of input neurons clearly decrease the MCUPS values.

**Figure 9:**
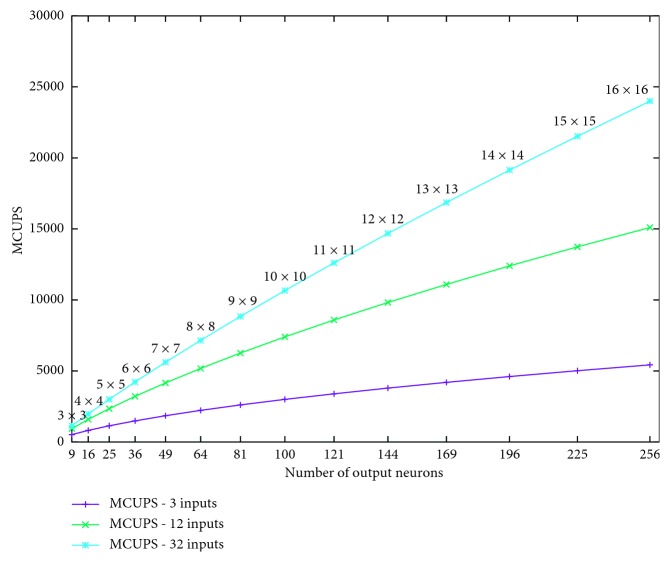
Variation in MCUPS according to the number of neurons on output matrix (3, 12, and 32 neurons on input layer).

Figure 9 depicts the MCUPS variations depending on the SOM network topology (*P* and *Q*) by considering a number of elements of an input vector variant from 3 to 32.

Most research on neural engineering aims to optimize these systems in order to accelerate their learning phase. This can be measured in terms of MCUPS, which contains several aspects related to the processing architecture and the network configuration.  Table 4 presents the experimental values compared to other studies that use maps of size almost similar to our SSOM network. The comparison is limited to the implantations that adopt a similar number of neural connections (which significantly affects the measurement of MCUPS) and use the same integration technology. For this last point, three different technologies (60 nm, 40 nm, and 28 nm) specific to three types of FPGA supports of the Xilinx family were used.

From the results of Table 4, it should be noted that the SSOM hardware architecture is relatively competitive compared to other more recent architectures. In addition, unlike parallel SOM architectures [8, 17, 18], SSOM is adaptive, based on the sequencing of the specific SOM operations. The latter will be performed concurrently in each neuroprocessor that will constitute the SOM network. Except the architecture proposed by Hikawa and Maeda in [24] which consumes a significant amount of resources, our architecture allows a relatively higher clock frequency and is highly flexible and configurable compared to the ones obtained in the literature. Noting that the obtained clock frequency is independent of the network size and input vector, which is not the case for the other hardware implementations shown in Table 3. Indeed, for SSOM networks with 256 output neurons and 32 inputs, we have achieved an MCUPS value between 1.4 and 5 faster than the one found in literatures which used the same technology as ours.

## 7. Application: Image Compression for SSOM Validation

To validate the SSOM, we propose the image compression based on the use of SOMs as an application. Indeed, the use of these networks for compression has been widely exploited in the literature [13, 16–18, 22–24]. Most of these models have been executed in three phases: color quantification, compressed image generation, and image reconstruction. Indeed, the vector quantification of colors is based on the use of SOM maps of *P* × *Q* dimensions. Thus, each neuron on the SOM map has a weight vector of the same size of one or more pixels (one pixel is represented by the three basic elements corresponding to the RGB colors). At the end of the learning phase, the weights of the neurons on the SOM card converge to stable values representing the colors that represent the image. As a consequence, the number of colors in an image is reduced according to the number of neurons available on the SOM card. Thus, a color palette is obtained by recovering the weights of neurons, called codebooks, at the end of the learning phase. This palette will be used during the compression and decompression phases of the image. Therefore, instead of using the actual color code to encode a pixel, we use the position of the neuron having the closest weights (color) to the color of the observed pixel, hence reducing its size.

Accordingly, starting from an original image of size *X* ∗ *Y* pixels the binary size of the image after compression is equal to *S*_c_ as follows:

(13) $$\begin{array}{c} S_{\text{c}}=X\cdot Y\cdot \left[\log_{2}\left(P\right)+\;\;\log_{2}\left(Q\right)\right]. \end{array}$$

We notice that the size of the image depends on the resolution of *P* and *Q* and the number of pixels of the original image. Let CR be the compression ratio of the image as follows:

(14) $$\begin{array}{c} \text{CR}=\left[1-\frac{S_{\text{c}}}{R\cdot X\cdot Y}\right]\cdot 100, \end{array}$$

where *R* represents the binary resolution of the weight vectors.

**Figure 10:**
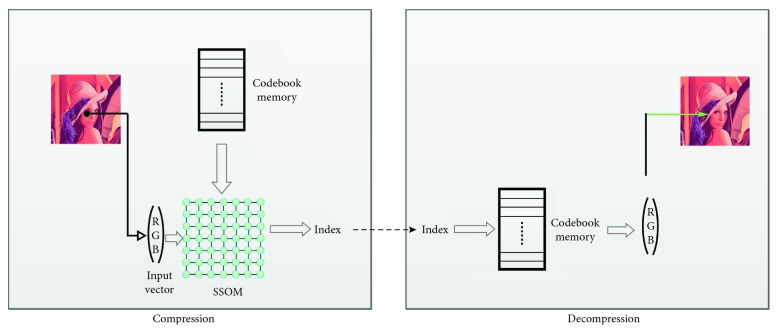
Image compression using SSOM architecture.

The principle of compression and decompression is illustrated in Figure 10.

The compression phase consists in rereading the original image pixel by pixel. Each pixel will then be presented to the SSOM network to extract the identifier of the winning neuron “index”. Each identifier, representing a part of the compressed image, will be saved. During the decompression phase, each of the identifiers of the compressed image will be used as a pointer to the codebook module, so as to retrieve its corresponding color. Once the scanning of the entire compressed image is performed, a reconstructed image will be recovered in order to be compared with the original image.

**Figure 11:**
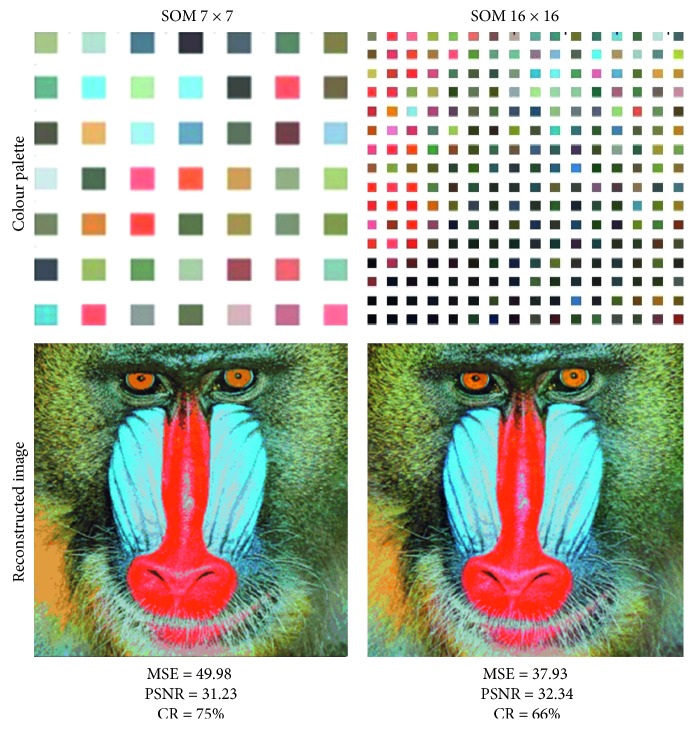
Quantization results for different configurations of Baboon image.

To validate our architecture, we use the color image (Figure 11). Indeed, this figure has two columns, and each one is specific to an SOM topology: 7 ∗ 7 and 16 ∗ 16. In each column, we represent the obtained results: the palette of quantized colors of the original image (codebook) whose size depends on the topology of the Kohonen map used, the reconstructed image, and the values of MSE, PSNR, and CR.

From the attained results obtained, we notice that the CR compression ratio depends exclusively on the topology of the chosen SOM network independently of the number of colors and the size of the original image to be compressed. Thus, the best CR is obtained with the topology of an SOM network formed by the smallest map at the output and the largest number of inputs (which reflects the number of pixels). However, the PSNR values (which reflect the visual quality) for each of the three images depend on the number of colors represented in the original image and on the topology used to quantify the colors of the image. Indeed, the PSNR value varies according to the number of neurons on the output layer of the SOM network (PSNR rises as the number of neurons goes up).

## 8. Conclusion

A massive parallel SOM neural network has been put forward. The proposed SOM network architecture, referred to as systolic-SOM (SSOM), has been described as a soft IP core synthesized in VHDL.

To implement the SSOM architecture, we have exploited parallel processing units, called NPs. The architecture of each NP is composed of distance calculation modules and weight updates, classically defined in most bibliographic work. It consists also of two other modules required respectively to extract the minimal distance and to calculate the neighbourhood function. The new NP architecture allowed us to give flexibility to our neural network independently of the design phase.

The SSOM architecture has been reinforced by generic formalism based on the graph theory, enabling their generalization in a flexible way for any modification or improvement. For SSOM networks with 256 output neurons and 32 inputs, we have achieved an MCUPS value twice that already obtained in the most recent literature.

In perspective, this same architecture could be adapted to a neural algorithm such as learning vector quantization (LVQ), which adopts the same training concept as the SOM with the only difference that it is supervised (it is mainly used for the classification). So to switch from SOM to LVQ, it is simply a matter of modifying the SOMPE' architecture (Figure 4) by removing the neighbourhood unit and adding an additional specific input to the expert's label that is necessary for supervised learning. This architecture may well be used in an electroencephalogram (EEG) classification application already published in [25] but adopting a different architectural approach.

## Figures and Tables

**Table 1:** Example of the systolic propagation of distances and identifiers with a 5 × 5 SOM.

	*d*_*l*,*k*_^2^, (*lk*): values of the minimum distances and the identifiers corresponding to the output of each neuron with coordinates (*l*, *k*)								
	*P*_1_	*P*_2_	*P*_3_	*P*_4_	*P*_5_	*P*_6_	*P*_7_	*P*_8_	*P*_9_
(*d*_0,0_^2^, Id_00_)^min^	23, (00)	22, (10)	5, (04)	5, (04)	5, (04)	4, (23)	4, (23)	4, (23)	2, (44)
(*d*_0,1_^2^, Id_01_)^min^	33, (01)	11, (02)	6, (03)	5, (04)	4, (23)	4, (23)	4, (23)	2, (44)	2, (44)
(*d*_0,2_^2^, Id_02_)^min^	11, (02)	6, (03)	5, (04)	4, (23)	4, (23)	4, (23)	2, (44)	2, (44)	2, (44)
(*d*_0,3_^2^, Id_03_)^min^	6, (03)	5, (04)	4, (23)	4, (23)	4, (23)	2, (44)	2, (44)	2, (44)	2, (44)
(*d*_0,4_^2^, Id_04_)^min^	5, (04)	5, (04)	5, (04)	4, (23)	2, (44)	2, (44)	2, (44)	2, (44)	2, (44)
(*d*_1,0_^2^, Id_10_)^min^	22, (10)	5, (20)	5, (20)	5, (20)	4, (23)	4, (23)	4, (23)	2, (44)	2, (44)
(*d*_1,1_^2^, Id_11_)^min^	90, (11)	22, (10)	5, (20)	4, (23)	4, (23)	4, (23)	2, (44)	2, (44)	2, (44)
(*d*_1,2_^2^, Id_12_)^min^	66, (12)	6, (13)	4, (23)	4, (23)	4, (23)	2, (44)	2, (44)	2, (44)	2, (44)
(*d*_1,3_^2^, Id_13_)^min^	6, (13)	4, (23)	4, (23)	4, (23)	2, (44)	2, (44)	2, (44)	2, (44)	2, (44)
(*d*_1,4_^2^, Id_14_)^min^	6, (14)	5, (04)	4, (23)	2, (44)	2, (44)	2, (44)	2, (44)	2, (44)	2, (44)
(*d*_2,0_^2^, Id_20_)^min^	5, (20)	5, (20)	5, (20)	4, (23)	4, (23)	4, (23)	2, (44)	2, (44)	2, (44)
(*d*_2,1_^2^, Id_21_)^min^	55, (21)	5, (20)	4, (23)	4, (23)	4, (23)	2, (44)	2, (44)	2, (44)	2, (44)
(*d*_2,2_^2^, Id_22_)^min^	44, (22)	4, (23)	4, (23)	4, (23)	2, (44)	2, (44)	2, (44)	2, (44)	2, (44)
(*d*_2,3_^2^, Id_23_)^min^	4, (23)	4, (23)	4, (23)	2, (44)	2, (44)	2, (44)	2, (44)	2, (44)	2, (44)
(*d*_2,4_^2^, Id_24_)^min^	22, (24)	4, (23)	2, (44)	2, (44)	2, (44)	2, (44)	2, (44)	2, (44)	2, (44)
(*d*_3,0_^2^, Id_30_)^min^	11, (30)	5, (20)	5, (20)	5, (20)	4, (23)	2, (44)	2, (44)	2, (44)	2, (44)
(*d*_3,1_^2^, Id_31_)^min^	10, (31)	10, (31)	5, (20)	4, (23)	2, (44)	2, (44)	2, (44)	2, (44)	2, (44)
(*d*_3,2_^2^, Id_32_)^min^	15, (32)	10, (31)	4, (23)	2, (44)	2, (44)	2, (44)	2, (44)	2, (44)	2, (44)
(*d*_3,3_^2^, Id_33_)^min^	16, (33)	4, (23)	2, (44)	2, (44)	2, (44)	2, (44)	2, (44)	2, (44)	2, (44)
(*d*_3,4_^2^, Id_34_)^min^	19, (34)	2, (44)	2, (44)	2, (44)	2, (44)	2, (44)	2, (44)	2, (44)	2, (44)
(*d*_4,0_^2^, Id_40_)^min^	23, (40)	11, (30)	5, (20)	5, (20)	2, (44)	2, (44)	2, (44)	2, (44)	2, (44)
(*d*_4,1_^2^, Id_41_)^min^	43, (41)	10, (31)	10, (31)	2, (44)	2, (44)	2, (44)	2, (44)	2, (44)	2, (44)
(*d*_4,2_^2^, Id_42_)^min^	12, (42)	12, (42)	2, (44)	2, (44)	2, (44)	2, (44)	2, (44)	2, (44)	2, (44)
(*d*_4,3_^2^, Id_43_)^min^	13, (43)	2, (44)	2, (44)	2, (44)	2, (44)	2, (44)	2, (44)	2, (44)	2, (44)
(*d*_4,4_^2^, Id_44_)^min^	2, (44)	2, (44)	2, (44)	2, (44)	2, (44)	2, (44)	2, (44)	2, (44)	2, (44)

**Table 2:** Parameters adopted for time performance evaluation.

Parameters	Values	Descriptions
$\vec{X}X_{w}$	8 bits	Accuracy of vector element at input
$\vec{M}M_{w}$	8 bits	Accuracy of element of node weight vector
*L*_*d*_	21 bits	Accuracy of distance
*P × Q*	Variable	SOM topology
Dim_*x*_	32	Dimension of input vector
Freq	290 MHz	Operation frequency

**Table 3:** Step times of parallel SOM in on-line learning.

Phase	Equation	Number of cycles (clk) SOM (*P* = *Q* = 16)
Calculating distance	*t*_d_ = Dim_*x*_ + 2	34
Propagation time	*t*_p_ = *P* + *Q* − 1	31
Competition time (location of neighbouring)	*t*_v_	1
Updating time	*t*_upd_ = Dim_*x*_ + 1	33
Decision time	*t*_c_ = *t*_d_ + *t*_p_	65
Weight adapting time	*t*_a_ = *t*_v_ + *t*_upd_	34
Learning time	*t*_l_ = *t*_c_ + *t*_a_	99

**Table 4:** Comparison of proposed SSOM architecture to previously published implementations.

Design	Size of the SOM (*P* × *Q*)	Dim_*x*_	Maximal frequency (MHz)	MCUPS	MCPS	Resources
[8] ASIC 65 nm	16 × 16	32	100	3,080	3,091	—
[17] FPGA 40 nm	256	64	79.8	—	21,845	—
[18] FPGA 28 nm	16 × 16	256	250	18,597	—	—
[24] FPGA 40 nm (virtex 6)	16 × 16	3	33	25,344	—	94,266 LUT
(SSOM) FPGA 60 nm	16 × 16	32	185	15,308	23,316	82,522 LUT
(SSOM) FPGA 40 nm			225	18,618	28,357	75,225 LUT
(SSOM) FPGA 28 nm			290	23,997	36,549	70,478 LUT

## Data Availability

The VHDL codes used to support the findings of this study are available from the corresponding author upon request.

## References

[1] Długosz R., Kolasa M., Bieliński K. Programmable triangular neighborhood function for Kohonen self-organizing map implemented on chip.

[2] Kolasa M., Długosz R., Pedrycz W. Problem of efficient initialization of large self-organizing maps implemented in the CMOS technology.

[3] Macq D., Verleysen M., Jespers P., Legat J.-D. (1993). Analog implementation of a Kohonen map with on-chip learning. *IEEE Transactions on Neural Networks*.

[4] Landolt O., Delgado-Frias J. G., Moore W. R. (1994). An analog CMOS implementation of a kohonen network with learning capability. *VLSI for Neural Networks and Artificial Intelligence*.

[5] Ruwisch D., Bode M., Purwins H.-G. (1993). Parallel hardware implementation of Kohonen’s algorithm with an active medium. *Neural Networks*.

[6] Hendry D. C., Duncan A. A., Lightowler N. (2003). IP core implementation of a self-organizing neural network. *IEEE Transactions on Neural Networks*.

[7] Porrmann M., Witkowski U., Rückert U. (2003). A massively parallel architecture for self-organizing feature maps. *IEEE Transactions on Neural Networks*.

[8] Kolasa M., Długosz R., Pedrycz W., Szulc M. (2012). A programmable triangular neighborhood function for a Kohonen self-organizing map implemented on chip. *Neural Networks*.

[9] Shi C., Yang J., Han Y. (2014). A 1000 fps vision chip based on a dynamically reconfigurable hybrid architecture comprising a PE array processor and self-organizing map neural network. *IEEE Journal of Solid-State Circuits*.

[10] An F., Zhang X., Chen L., Mattausch H. J. (2016). A memory-based modular architecture for SOM and LVQ with dynamic configuration. *IEEE Transactions on Multi-Scale Computing Systems*.

[11] Porrmann M., Witkowski U., Rückert U., Omondi A., Rajapakse J. (2006). Implementation of self-organizing feature maps in reconfigurable hardware. *FPGA Implementations of Neural Networks*.

[12] Tamukoh H., Sekine M. A dynamically reconfigurable platform for self-organizing neural network hardware.

[13] Ramirez-Agundis A., Gadea-Girones R., Colom-Palero R. (2008). A hardware design of a massive-parallel, modular NN-based vector quantizer for real-time video coding. *Microprocessors and Microsystems*.

[14] Dlugosz R., Kolasa M., Szulc M. An FPGA implementation of the asynchronous programmable neighborhood mechanism for WTM selforganizing map.

[15] Kurdthongmee W. (2010). Utilization of a fast MSE calculation approach to improve the image quality and accelerate the operation of a hardware K-SOM quantizer. *Microprocessors and Microsystems*.

[16] Kurdthongmee W. (2016). A hardware centric algorithm for the best matching unit searching stage of the SOM-based quantizer and its FPGA implementation. *Journal of Real-Time Image Processing*.

[17] Huang Z., Zhang X., Chen L. (2017). A hardware-efficient vector quantizer based on self-organizing map for high-speed image compression. *Applied Sciences*.

[18] Abadi M., Slavisa J., Ben Khalifa K., Weber S., Bedoui M. H. (2018). A scalable and adaptable hardware NoC-based self organizing map. *Microprocessors and Microsystems*.

[19] Ienne P., Thiran P., Vassilas N. (1997). Modified self-organizing feature map algorithms for efficient digital hardware implementation. *IEEE Transactions on Neural Networks*.

[20] Manolakos I., Logaras E. High throughput systolic SOM IP core for FPGAs.

[21] Kohonen T. (2003). *Self-Organization Maps*.

[22] Pei S. C., S Lo Y. (1998). Colour image compression and limited display using self-organization Kohonen map. *IEEE Transactions on Circuits and Systems for Video Technology*.

[23] Chang C. H., Shibu M., Xiao R., Omondi A., Rajapakse J. (2006). Self organizing feature map for color quantization on FPGA. *FPGA Implementations of Neural Networks*.

[24] Hikawa H., Maeda Y. (2015). Improved learning performance of hardware self-organizing map using a novel neighborhood function. *IEEE Transactions on Neural Networks and Learning Systems*.

[25] Blaiech A. G., Ben Khalifa K., Boubaker M., Bedoui M. H. (2018). LVQ neural network optimized implementation on FPGA devices with multiple-wordlength operations for real-time systems. *Neural Computing and Applications*.

